# Environmental Controls on Crenarchaeol Distributions in Hydrothermal Springs

**DOI:** 10.1111/1462-2920.70248

**Published:** 2026-02-16

**Authors:** Amanda N. Calhoun, Jerome Blewett, Daniel R. Colman, Maximiliano J. Amenabar, Carolynn M. Harris, Eric S. Boyd, Ann Pearson, William D. Leavitt

**Affiliations:** ^1^ Department of Earth Sciences Dartmouth College Hanover New Hampshire USA; ^2^ Department of Earth & Planetary Sciences Harvard University Cambridge Massachusetts USA; ^3^ Department of Microbiology & Cell Biology Montana State University Bozeman Montana USA; ^4^ Escuela de Biotecnología, Facultad de Ciencias Universidad Santo Tomás Santiago Chile; ^5^ Department of Geology & Geophysics University of Utah Salt Lake City Utah USA; ^6^ Department of Chemistry Dartmouth College Hanover New Hampshire USA

**Keywords:** aqueous geochemistry, archaeal lipids, biomarkers, thermal springs

## Abstract

Thermophilic archaea synthesise cellular membranes composed primarily of isoprenoid glycerol dibiphytanyl glycerol tetraethers (iGDGTs). Cells can adjust the packing of their lipids by increasing cyclopentyl ring production, thereby decreasing membrane permeability and fluidity to maintain cellular function at high temperature, acidic pH, or nutrient limitation. Archaea of the class *Nitrososphaeria* synthesise crenarchaeol, an iGDGT with four cyclopentyl rings and a cyclohexyl ring, the function of which is unknown. Structural modelling suggests the cyclohexyl ring may increase membrane fluidity, potentially optimising membranes for mesophilic conditions. To investigate the role of crenarchaeol in archaeal membranes in natural settings, we quantify iGDGT compositions of forty‐one thermal springs in Yellowstone National Park (YNP), USA, and contextualise these within a global compilation of thermal spring iGDGTs spanning pH values of 1.1–10.1 and temperatures of 16°C–95°C. Spring pH is the strongest predictor of both crenarchaeol relative abundance and the number of cyclopentyl rings per iGDGT. Crenarchaeol relative abundance exhibits a nonlinear relationship with pH and temperature, with highest relative abundances at pH 7.4 and 46°C, decreasing above and below these values. These observations indicate that the cyclohexyl ring of crenarchaeol optimises archaeal cellular membranes for circumneutral and moderate temperature environmental conditions.

## Graphical abstract

The optimal pH and temperature for crenarchaeol (a unique archaeal lipid) production in hydrothermal springs are 7.4°C and 46°C, respectively, which has important implications for archaeal evolution. Our study also indicates that pH is the most important environmental variable for archaeal lipid membrane compositions.

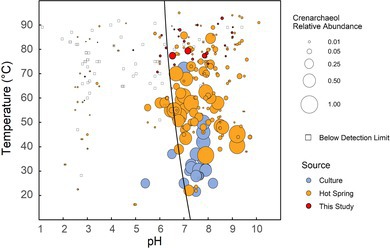

## 1 Introduction

Terrestrial hydrothermal systems are valuable analogues of early Earth environments where many origin(s) of life theories suggest life first emerged due to sharp thermal and geochemical gradients (Damer and Deamer 2020). Archaea are ubiquitous in these environments and are adapted to the extreme conditions within these systems (van de Vossenberg et al. 1998; Macalady et al. 2004) that impose chronic energy stress (Valentine 2007). A primary adaptation of archaea that inhabit extreme geothermal environments is the structural properties of their isoprenoid glycerol dibiphytanyl glycerol tetraether (iGDGT) lipids that comprise their cell membranes (van de Vossenberg et al. 1998; Konings et al. 2002). Archaeal iGDGTs are also ubiquitous in cells that inhabit soils, lakes, marine waters, and sediments, where they can serve as paleoenvironmental proxies (Schouten et al. 2000, 2002, 2013). Interactions between archaea and their environments have shaped their adaptive evolution (Colman et al. 2018; Yang et al. 2021), specifically the ability to adjust cell membrane fluidity and permeability in response to physical and chemical stressors (Oger and Cario 2013).

**FIGURE 1:**
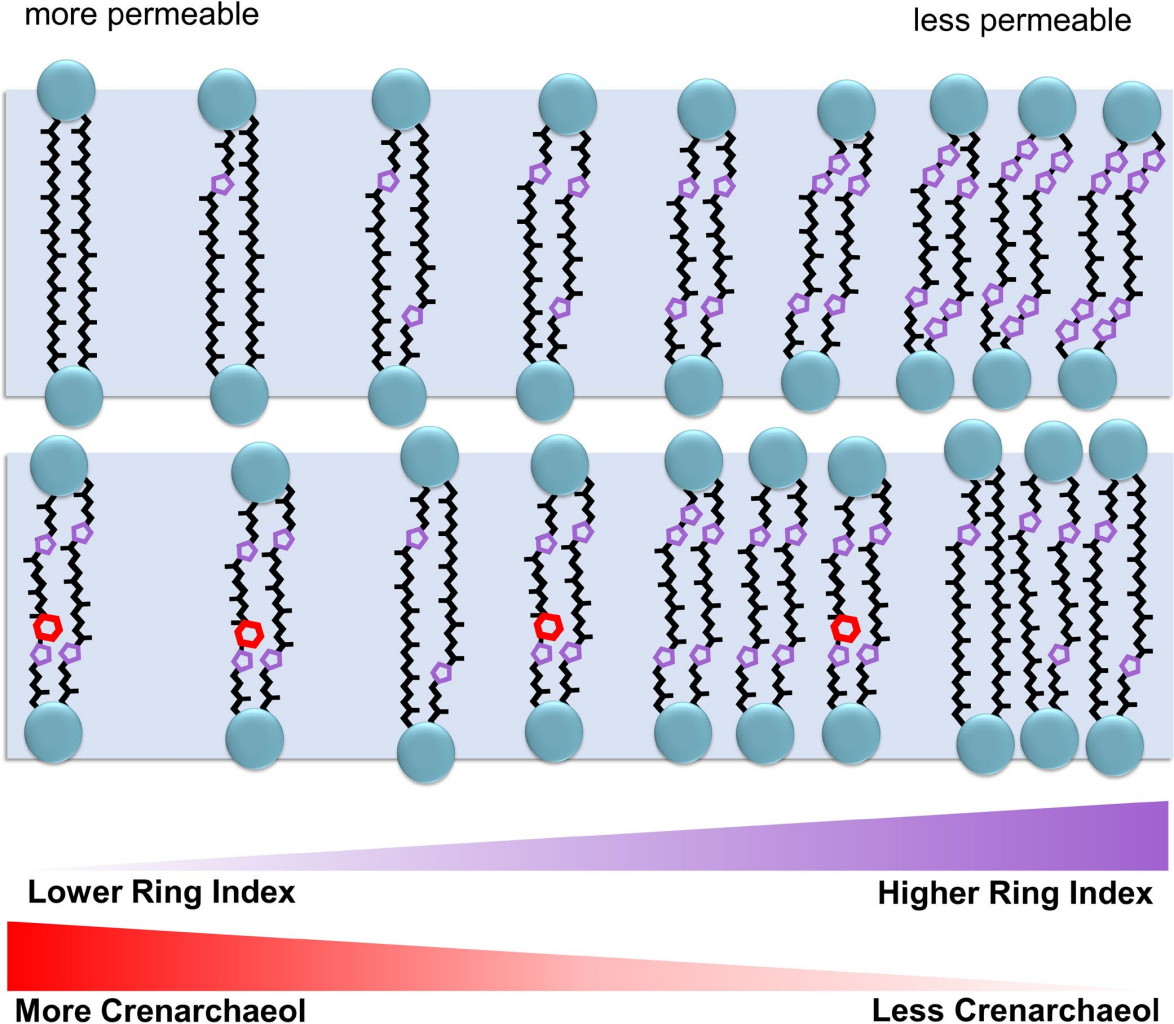
Schematic of effects of cyclopentyl rings (purple) on archaeal membrane permeability versus the hypothesised effect of cyclohexyl rings (red) on archaeal membrane permeability, indicated by lower bars.

The membranes of thermophilic archaea are primarily composed of iGDGTs (Macalady et al. 2004; Oger and Cario 2013). These molecules vary in the number of cyclopentane or cyclohexane rings present in their internal biphytanyl chains (De Rosa et al. 1980; De Rosa and Gambacorta 1988; Schouten et al. 2000). The rings in iGDGTs affect molecular packing (Figure 1), enabling the modulation of archaeal cell membrane fluidity and permeability in response to changing temperature, pH, energy stress, salinity, and pressure through production of more or fewer rings as needed (Albers et al. 2000; Gabriel and Lee Gau Chong 2000; Boyd et al. 2011; Pearson and Ingalls 2013; Feyhl‐Buska et al. 2016; Zhou et al. 2020). In most environments, archaea produce iGDGTs with zero to four cyclopentane rings (iGDGT‐0 to iGDGT‐4), but in thermal springs, archaea can produce iGDGTs with up to eight cyclopentane rings (iGDGT‐8) (Pancost et al. 2006; Schouten et al. 2013). The increase in average ring counts of iGDGTs at higher temperatures is the basis for the TEX_86_ (TetraEther indeX of 86 carbon atoms) paleotemperature proxy (Schouten et al. 2002), which is used to infer ancient environmental temperatures. Recent molecular dynamics simulations of iGDGTs across variable temperatures have also supported a temperature control of archaeal lipid distributions over geologic timescales (Zhao et al. 2025). However, recent studies indicate that additional environmental parameters can influence cyclopentyl ring abundance, complicating the interpretation of TEX_86_ as a strict temperature proxy (Qin et al. 2015; Elling et al. 2015; Hurley et al. 2016; Zhou et al. 2020; Cobban et al. 2020; Tourte et al. 2022).

Crenarchaeol iGDGT lipids and associated isomers (Sinninghe Damsté et al. 2018) are unique amongst iGDGTs in that they contain a cyclohexane ring (Sinninghe Damsté et al. 2002). Crenarchaeol is so far specific to representatives of the class *Nitrososphaeria* (syn. *Thaumarchaeota*) that are ubiquitous in terrestrial thermal springs, soils, and marine water columns, where they can be the most abundant nitrifiers (Ueda et al. 1995; Karner et al. 2001; Stahl and de la Torre 2012). At present, the origin and biosynthetic mechanism of this ring remain unknown, whilst hypotheses on its physiological function are not yet well‐established (Zhang et al. 2006). Whilst recent work has revealed many steps of iGDGT synthesis (e.g., Zeng et al. 2022; Lloyd et al. 2022), the biosynthetic pathway of iGDGT cyclohexyl rings remains elusive, leaving the natural history and function of crenarchaeol unknown. In addition to crenarchaeol, organisms of the *Nitrososphaeria* produce iGDGT‐0 to iGDGT‐4, some of which contribute to TEX_86_ calculations. In thermal springs, the often‐abundant *Nitrososphaeria* contains the only subgroup demonstrated to produce crenarchaeol, the ammonia‐oxidising archaea (AOA; De La Torre et al. 2008; Pitcher et al. 2010; Boyd et al. 2013). The crenarchaeol‐producing AOA are thought to have originated as thermophilic descendants of non‐AOA *Nitrososphaeria* (Abby et al. 2020; Luo et al. 2024). The predecessors of extant crenarchaeol‐producing AOAs would have expanded their habitat from thermal springs to cooler marine waters between 629 and 412 Ma based on phylogenomic and marker gene (*amoA*) analysis of environmentally diverse archaeal genomes (Yang et al. 2021). As such, the progenitor that was originally optimised to live at high temperatures had to adapt its membrane structure away from the dense, rigid membranes of thermophilic archaea with high numbers of cyclopentyl rings. Incorporating the cyclohexyl ring into iGDGTs (i.e., crenarchaeol) may have relaxed the tight molecular packing in thermophile cellular membranes, thus facilitating the colonisation of AOA progenitors in the less ‘extreme’ marine realm (Schouten et al. 2000; Figure 1). This hypothesis is supported by lipid modelling that indicates the cyclohexane ring forms a bulge that increases biphytane volume, preventing dense membrane packing (Sinninghe‐Damsté et al. 2002). However, recent molecular dynamics simulations indicate that the inclusion of a cyclohexane ring may in fact decrease membrane fluidity but increase permeability (Zhou and Dong 2025), suggesting an adaptation to high temperatures or predominant control by variables other than temperature. A previous environmental survey suggested a 40°C temperature optimum for crenarchaeol given its abundance normalised to GDGT‐0 in marine sediments and thermal spring microbial mats (Zhang et al. 2006), though crenarchaeol can be the most abundant core lipid in the membrane of cultured archaea above 70°C (De La Torre et al. 2008). Whilst temperature is known to influence the membrane lipid composition of marine archaea, a clear negative correlation between crenarchaeol abundance and acidity in thermal spring archaeal communities (Boyd et al. 2013) implies that crenarchaeol is synthesised to a greater extent in circumneutral waters as opposed to acidic conditions. These competing predictions and observations motivate the current study to better understand whether temperature is the major determinant of environmental crenarchaeol distributions.

Here we utilise lipid data to examine patterns of crenarchaeol relative abundance and distribution from 299 thermal spring samples from North America, Europe, and Asia that span variable temperature (16°C–95°C), pH (1.1–10.1), redox (−330 to 330 mV for a new Yellowstone National Park 41 sample subset), and other geochemical conditions. In parallel, we calculate the summary indicator of cyclopentyl ring abundance, Ring Index (RI). This approach enables us to re‐evaluate potential archaeal adaptations to environmental stress, allowing for the examination of key geochemical factors that drive crenarchaeol abundance on the present and past Earth.

## 2 Procedures

### 2.1 Field Measurements and Sample Collection

**FIGURE 2:**
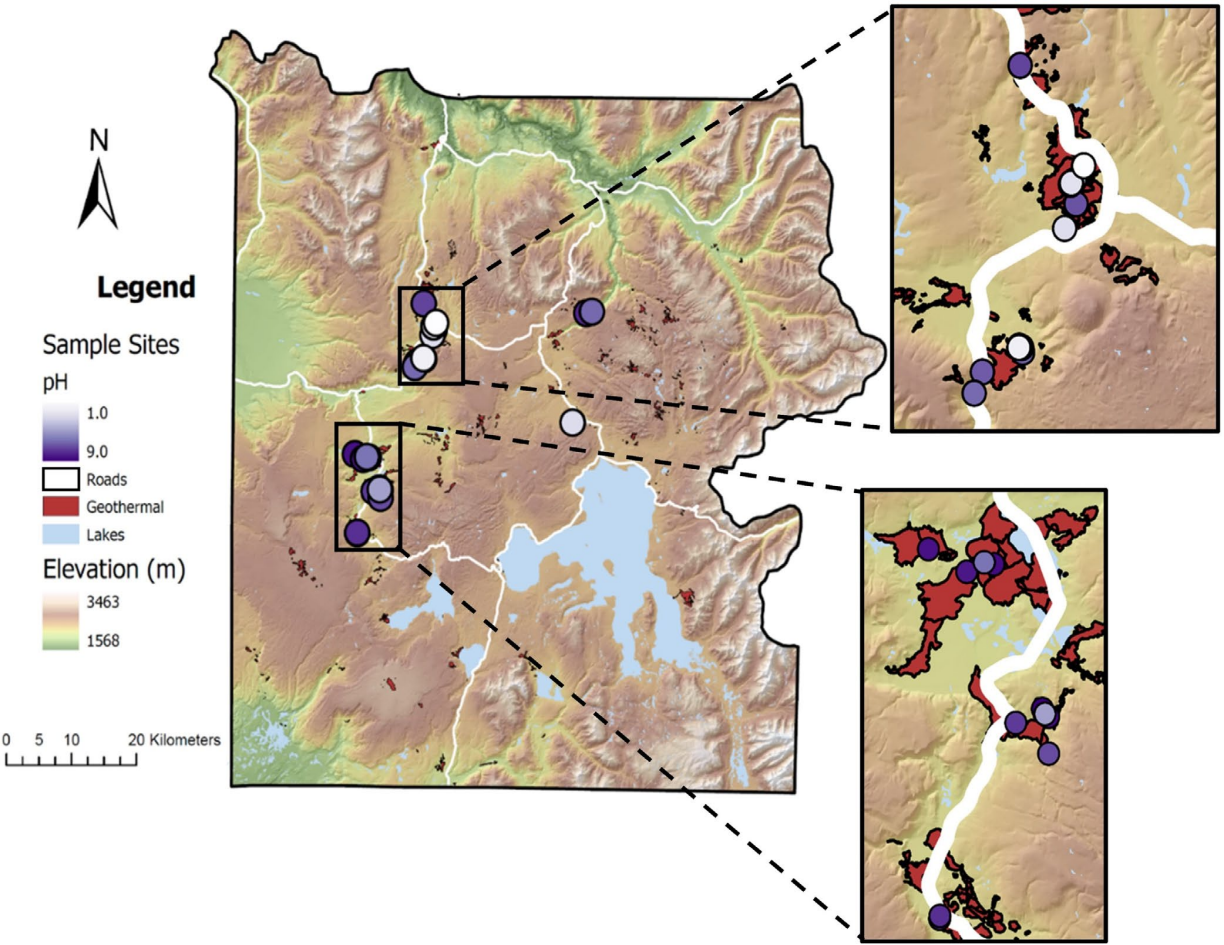
Map of sampling locations coloured by pH in Yellowstone National Park, USA 2018 to 2022. Major roads are shown in white and geothermal areas are shaded in red.

A total of 41 sediment samples, encompassing 38 individual springs, were collected over four field seasons between 2018 and 2022 in Yellowstone National Park, USA (permit #YELL‐05544) (Figure 2). Water temperature (°C), pH, dissolved oxygen (DO) concentration, specific conductivity (SPC), and oxidation reduction potential (ORP) were measured in the field at the time of sampling using a YSI ProDSS metre calibrated for DO and with pH buffer solutions of 4, 7, and 10 or 1.68, 4, and 7 (YSI, Yellow Springs, OH, USA). Concentrations of dissolved sulphide (ΣH_2_S/HS^−^/S^2−^; detection limit 5 μg/L) and Fe (II; detection limit 0.01 mg/L) were determined in the field with a portable Hach spectrophotometer (model DR2800) and Hach reagents (Hach Company, Loveland, CO, USA) following established protocols (Colman et al. 2016). Sulphide and Fe (II) concentrations were measured on water samples collected by a high‐density polyethylene sampling staff (turbid water samples were filtered through pre‐sterilised 0.22 μm Sterivex filters (EMD Millipore, Billerica, MA, USA)), whilst all other parameters were measured in situ near the site of sediment collection. Sediments were collected into sterile conical centrifuge tubes using the sampling staff, placed on dry ice in the field, then transferred to storage at −80°C until further processing.

### 2.2 Lipid Extraction and Preparation

Sediment samples were freeze‐dried and stored at room temperature before extraction. Total lipid extracts (TLEs) were obtained by modified Bligh‐Dyer extraction (Bligh and Dyer 1959; Weber et al. 2017). Sediments (~1 g) were sonicated in 2:1:0.8 methanol:dichloromethane:trichloroacetic acid (MeOH:DCM:TCA) buffer (TCA, 50 g/L, pH = 2.1), centrifuged, and phase‐extracted in four rounds. Organic phases underwent a final water rinse before concentration under N_2_ gas and reconstitution in 2:1 DCM:methanol. Where necessary, multiple samples were merged for larger lipid yields (Table S1). TLEs were treated with activated (1 M HCl, rinsed with H_2_O, MeOH, DCM, and hexane) copper at room temperature overnight to remove S^0^ (Boyd et al. 2011). TLEs were separated into core lipid (CL) and intact polar lipid (IPL) fractions by solvent elution through SiO_2_ columns as described in Pitcher et al. (2009) and stored in DCM at −20°C. The polar headgroups of IPL fractions were cleaved (5% HCl in MeOH, 3 h). IPL‐derived lipids were brought to a pH of 4–5 using 1 M KOH in MeOH, followed by four rounds of liquid–liquid extraction. CL‐ and IPL‐derived lipid fractions were reconstituted in a mixture of 99:1 hexane:isopropyl alcohol (IPA) with addition of 100 ng of C_46_‐GTGT internal standard (Huguet et al. 2006). All samples were filtered through 0.45 μm PTFE filters (4 mm diameter) before analysis.

### 2.3 iGDGT Identification and Relative Quantification

All iGDGT fractions were analysed by ultra‐high performance liquid chromatography—atmospheric pressure chemical ionisation—mass spectrometry (UHPLC‐ACPI‐MS) using an Agilent 1290 Infinity series UHPLC system coupled to an Agilent 6460 triple‐quadrupole mass spectrometer (QQQ MS), consistent with methods used in earlier work (Zhou et al. 2020; Blewett et al. 2020). GDGTs were separated by injecting 10 μL of sample onto two coupled ACQUITY UPLC BEH Amide Columns (1.7 μm, 2.1 × 150 mm) held at 50°C (Becker et al. 2013) with a constant solvent flow rate (0.5 mL/min) and solvent mixtures A (pure hexane), B (90:10 hexane:IPA), and D (30:70 MeOH:IPA). The programme started at 98:2 A:B with a linear gradient to 3% B by 4 min, 10% B by 10 min, 20% B by 20 min, 50% B by 35 min, and 100% B by 40 min followed by a 1‐min hold. At 41.01 min, the programme was set to 70:30 B:D, ramping to 100:0 B:D by 46 min and 98:2 A:B by 47 min, holding until the total run time of 65 min. The QQQ‐MS was operated in single ion monitoring (SIM) mode with a dwell time of 128 ms and fragmentor voltage of 75 V. GDGT relative abundances were determined by manual integration of ion chromatograms with mass to charge ratios (*m*/*z*) starting at 1302.3 for iGDGT‐0 and decreasing by increments of 2 down to 1286.3 *m*/*z* for iGDGT‐8.

### 2.4 Data Reduction and Statistical Analyses

Two RI values were calculated: with and without crenarchaeol [Equations (1 and 2)]. In the numerator, the relative abundance of each lipid is multiplied by the number of cyclopentyl rings in the lipid structure (Taylor et al. 2013; Zhang et al. 2016). For this reason, crenarchaeol is incorporated as a four‐ringed member in Equation (1) because the fifth ring is the enigmatic cyclohexyl ring whose contribution to membrane dynamics is so far unconstrained (Zhang et al. 2016). See the Supplement for a discussion of the merits and drawbacks of including crenarchaeol as a four‐ringed contributor to RI; we conclude that it is appropriate to exclude crenarchaeol from RI calculations if the intended purpose is to investigate membrane permeability.

(1) $$\begin{array}{c} {\mathrm{RI}}_{\left(+\text{cren}\right)}=\left[\frac{G1+2*G2+3*G3+4*\left(G4+\text{Cren}+\mathrm{Cre}n^{\prime }\right)+5*G5+6*G6+7*G7+8*G8}{G0+G1+G2+G3+G4+\text{Cren}+\mathrm{Cre}n^{\prime }+G5+G6+G7+G8}\right] \end{array}$$

(2) $$\begin{array}{c} {\mathrm{RI}}_{\left(-\text{cren}\right)}\\ =\left[\frac{G1+2*G2+3*G3+4*G4+5*G5+6*G6+7*G7+8*G8}{G0+G1+G2+G3+G4+G5+G6+G7+G8}\right] \end{array}$$

Given that neutral pH varies with temperature, we calculated the temperature‐dependent neutral pH, i.e., the neutrality line, from 0°C to 100°C [Equation (3); Justnes 2020).

(3) $$\text{Neutral}\;\mathrm{pH}=8*\left(T^{2}*10^{-5}\right)-\left(0.0208*T\right)+7.4692$$

Statistical analyses were performed using R Statistical Software (v4.4.0; R Core Team 2024). Scripts for statistical runs and plots are available at https://zenodo.org/doi/10.5281/zenodo.12603497. Quadratic and interaction terms were tested in modelling, but these were not substantially more powerful than reported models. All input variables of regression models are normalised using the base *scale*() function of R to prevent skew due to parameter magnitude.

## 3 Results

### 3.1 Lipid Characteristics Amongst YNP Hydrothermal Springs

Geochemical parameters for the YNP samples collected in this study (*n* = 41; Table S1) include temperature (28°C–93°C), pH (1.1–9.0), dissolved oxygen (DO; 1.6 × 10^−7^ to 6.2 × 10^−5^ M), specific conductivity (SPC; 50 to 5380 μS/cm), oxidation reduction potential (ORP; −330 to 330 mV), dissolved Fe (II) (B.D. to 3.5 × 10^−5^ M) and dissolved sulphide (S^2−^; B.D. to 2.5 × 10^−4^ M) concentrations. As none of these parameters were normally distributed, Spearman's rho correlations were used to evaluate their relationships with lipid characteristics. The relationships between these environmental variables, crenarchaeol relative abundance, and Ring Index (see Table S1 for individual site values) were calculated using simple (SLR) and multiple linear regressions (MLR) of all possible geochemical parameter combinations for both CL and IPL fractions. Model results reported below are for CL fractions and statistical results for both fractions are compared in Tables S2, S3, S5, S6, as CL lipids are often more abundant and represent a more integrative record of iGDGT production than IPL lipids. The RI results reported here include crenarchaeol as a four‐ringed member, as seen in previous studies (Zhang et al. 2016).

The non‐linear Spearman's rho tests demonstrated that pH and Fe (II) concentration were the only variables that were significantly associated with CL and IPL crenarchaeol relative abundance for the 41 new YNP samples (Table S2). However, significant relationships between Fe (II) concentration and crenarchaeol relative abundance are likely due to the strong correlation of Fe (II) with pH (Figure S6; Amenabar and Boyd 2018), with the latter being the likely true driver of crenarchaeol relative abundance. None of the individual environmental variables exhibited significant (*p* < 0.05) simple linear regressions with crenarchaeol relative abundance, but several combinations of variables exhibit significant multiple linear regression relationships with crenarchaeol (Table S3). Of all possible variable combinations (127 models), the most important environmental parameters for predicting crenarchaeol relative abundance were pH, SPC, and ORP. All five multiple linear regression models with the highest adjusted *R* ^2^ values include these three variables, which alone explain 19% of the variance (adj‐*R* ^2^ = 0.19) (Table S4). The model that incorporates sulphide as a fourth variable in addition to pH, SPC, and ORP is the best overall predictor (adj‐*R* ^2^ = 0.21). In contrast, including DO or temperature as the fourth variable decreases the model explanatory power (adj‐*R* ^2^ = 0.17).

Spearman's rho tests of RI values showed that pH, ORP, and Fe (II) concentration were significantly associated with both the CL and IPL fractions from the YNP thermal spring samples (Table S5). Only the CL fraction had a non‐linear association with temperature. Simple linear regression results matched those of the Spearman's rho correlation, with pH, ORP, and Fe (II) concentration correlating with both CL and IPL fractions, whilst temperature only correlated with the CL fraction (Table S6). Sulphide has a significant linear relationship with IPL‐RI, but there is no non‐linear association (Spearman's) between these two variables. Of all 127 possible multiple linear regression models of RI, the ten most significant (*p* < 0.05) models include pH and DO, whilst Fe (II) is present in eight, temperature in six, sulphide in five, SPC in three, and ORP in two (Table S7). The best explanatory models incorporate pH, temperature, DO, and Fe (II) (adj‐*R* ^2^ = 0.80), or just the three variables pH, DO, and Fe (II) (adj‐*R* ^2^ = 0.79). The addition of a fourth variable other than temperature slightly decreases model predictive power, but all ten most significant models exhibit an adjusted *R* ^2^ range of 0.79–0.80. In high temperature hot springs that exclude photosynthesis, the availability of DO is controlled, at first order, by temperature‐dependent O_2_ solubility (Shock et al. 2010), indicating that temperature may be the real driver of crenarchaeol relative abundance in cases above in which DO is implicated as an important variable.

### 3.2 Comparison to Previously Published Studies

**FIGURE 3:**
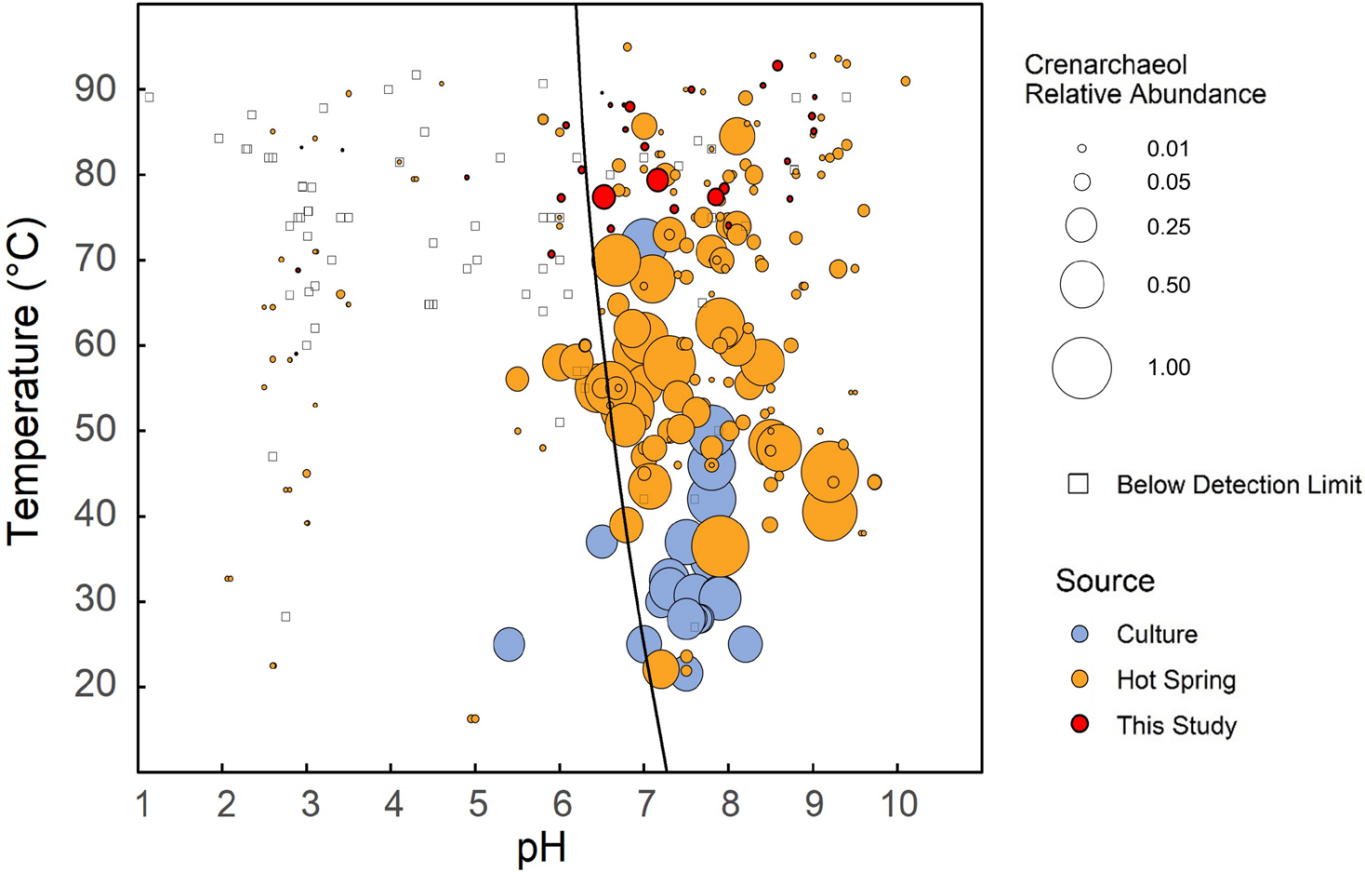
Relative abundances of crenarchaeol for all compiled thermal spring samples (*N* = 299) and culture samples (*n* = 33). Samples with no detectable crenarchaeol are represented by squares and those with crenarchaeol present are circles filled by the source of the data. Area of circles correlates with crenarchaeol relative abundance. The black curve is the temperature‐dependent neutral pH line (Equation 3).

We compiled published thermal spring lipid samples (*N* = 299 including 41 from this study) that report pH, temperature, and iGDGT abundances, specifically including crenarchaeol abundance (Pearson et al. 2004, 2008; Zhang et al. 2006; Schouten et al. 2007; Pitcher et al. 2009; Zhao et al. 2011; Burgess et al. 2012; He et al. 2012; Li et al. 2013; Boyd et al. 2013; Wu et al. 2013; Paraiso et al. 2013; Jia et al. 2014; Xie et al. 2015). We include relevant culture data in Figure 3 for context but exclude this data from our statistical analyses to focus on environmental samples. For previous studies that split iGDGTs into CL and IPL fractions, the CL crenarchaeol relative abundances were used in this compilation since CL‐iGDGTs are generally dominant and represent a more integrative record of iGDGT production than IPL lipids. The compiled dataset affords greater environmental context than the dataset from YNP samples alone; however, it reduces the available environmental variables to pH and temperature, which we focus on in our interpretation.

Across all sites, crenarchaeol relative abundances were highest to the alkaline side (≥ 7.0 pH) of the temperature‐dependent neutral pH line (Equation 3), whilst acidic (< 7.0 pH) springs frequently have non‐detectable levels of crenarchaeol (Figure 3). The 109 acidic samples have an average fractional crenarchaeol abundance of 0.02 (2% of all iGDGTs or CL iGDGTs), whilst that of the 190 alkaline samples is 0.10, with many examples > 0.50. Crenarchaeol was undetectable in 35 of 109 (32.1%) acidic samples versus 12 of 190 (6.3%) of alkaline samples. Crenarchaeol relative abundance also shows a weak relationship to temperature. Of 201 samples from environments ≥ 60°C, 79.6% have detectable crenarchaeol. Ninety‐eight samples are from < 60°C, of which 93.9% contain detectable crenarchaeol. Additionally, at ≥ 60°C, the average crenarchaeol fractional abundance is 0.04 (0.05 without zero values) and < 60°C, the average is 0.14 (0.15 without zeroes).

### 3.3 Statistical Analyses

Simple and multiple linear regression models were conducted to evaluate the influence of pH, temperature, and the combination of both variables for the full dataset (Table 1). MLR models incorporating interactions between temperature and pH and quadratic variables were run, but did not noticeably improve *R* ^2^ values (0.13 with interactions and 0.15 with quadratic variables for the crenarchaeol models), so linear models are shown for simplicity. Non‐linear Spearman's rho correlations indicated that crenarchaeol is strongly associated with both pH and temperature (*p* = 1 × 10^−13^ and 5 × 10^−9^, respectively), whilst Ring Index (Equation 1) is associated with pH (*p* = 7 × 10^−9^) but not with temperature (*p* = 0.8). Whilst all *R* ^2^ values show relatively low explanatory power, pH dominates predictions of RI, whilst both pH and temperature are significant individual predictors of crenarchaeol relative abundance.

**TABLE 1:** Outputs of simple and multiple linear regression models for pH and temperature relationships with crenarchaeol relative abundance and Ring Index of all compiled data (*N* = 299).

	Parameter(s)	Crenarchaeol		RI	
		*p*	adjusted *R* ^2^	*p*	adjusted *R* ^2^
Simple linear regression	pH	**< 0.001**	0.033	**< 0.0001**	0.15
	Temp (°C)	**< 0.0001**	0.073	0.62	−0.0025
Multiple linear regression	pH + Temp (°C)	**< 0.0001**	0.11	**< 0.0001**	0.15

*Note:* Adjusted *R* ^2^ values are reported, and significant *p*‐values are indicated by bold italics. Note that negative *R* ^2^ values indicate a fit worse than a horizontal line.

**FIGURE 4:**
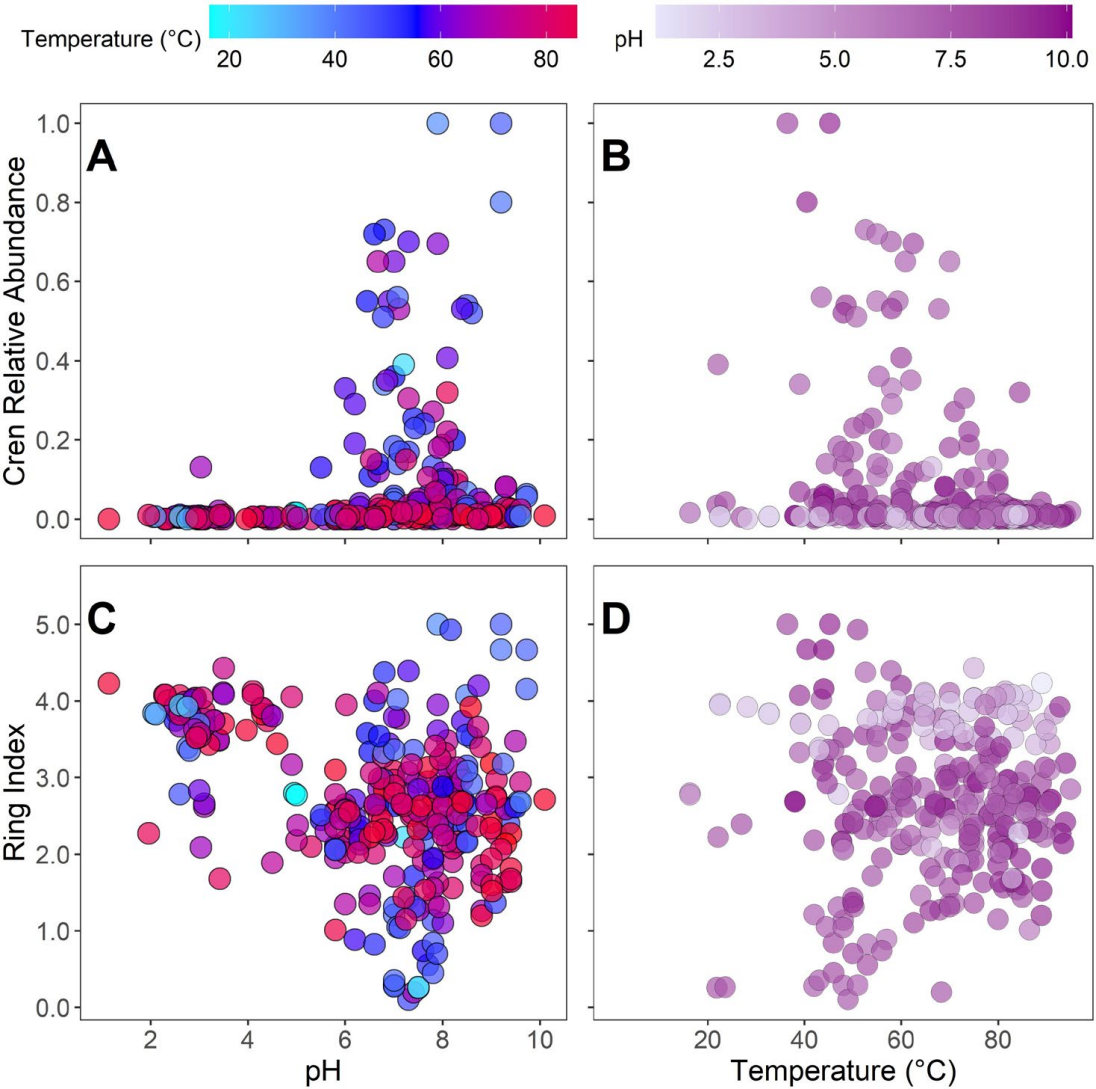
Crenarchaeol relative abundance versus (A) pH and (B) temperature for the 299 compiled samples, with fill by the other variable. Ring Index versus (C) pH and (D) temperature for the 299 compiled samples, with fill by the other variable.

The two variables are visualised in Figure 4. Relative crenarchaeol abundance is lower in many high‐temperature samples and lower or absent in most of the lowest‐pH samples (Figure 4A,B). RI has no apparent patterns associated with temperature, but high RI values are often identified in low‐pH samples (Figure 4C,D). Whilst the linear regression models are statistically significant, all these correlations are modest (Table 1), and some of the highest RI values occur at high pH values (e.g., Figure 4C).

## 4 Discussion

### 4.1 Environmental Predictors of Ring Index

Studies of marine systems generally consider temperature to be the primary driver for iGDGT cyclization (Schouten et al. 2002; Kim et al. 2008, 2010; Tierney 2012), which may result from the relative geochemical uniformity of modern open‐ocean environments. In marine waters, deviations from global average RI or TEX_86_ vs. temperature correlations are associated with pH, DO concentration, ammonia oxidation rate, growth phase, and salinity (Elling et al. 2014; Qin et al. 2015; Elling et al. 2015; Hurley et al. 2016, 2018), demonstrating that variables other than temperature may be of greater importance in other settings (Boyd et al. 2011; Elling et al. 2015; Cobban et al. 2020). It is likely that control of iGDGT cyclization in thermal springs is different from the marine system because thermal springs generally span larger gradients of environmental variables, including but not limited to pH. Previous studies examining iGDGT cyclization in thermal springs have yielded conflicting conclusions. Temperature, pH, salinity, redox potential, bicarbonate concentration, growth rate, and DO all have been reported to correlate with iGDGT cyclization (Pearson et al. 2004; Boyd et al. 2013; Elling et al. 2014, 2015; Qin et al. 2015; Feyhl‐Buska et al. 2016; Evans et al. 2018; Cobban et al. 2020; Zhou et al. 2020). Whilst the degree of importance of each of these variables on iGDGT cyclization has not been definitively established, some studies conclude that pH has a greater influence than temperature. For example, thermal springs spanning pH 5.5–7.2 in New Zealand showed increased RI at higher temperatures, but more acidic springs (pH 2.1–5.5) did not demonstrate this temperature effect (Kaur et al. 2015). These results indicate that acidity is more important than temperature for affecting archaeal membrane composition, potentially in direct response to pH‐induced stress. The discrepancies amongst studies may be due to sampling relatively narrow ranges of geochemical and geophysical parameters, or it may result from the competing effects of several environmental factors (e.g., temperature and pH; Pearson et al. 2008; Wu et al. 2013; Boyd et al. 2013; Xie et al. 2015). We synthesise prior works and our new observations from YNP to consider the environmental stressors of pH and temperature together, rather than in isolation.

Here, our analysis of compiled global thermal springs (*N* = 299) demonstrates that pH significantly influences RI, whilst temperature does not. Linear regression models for the 41 YNP samples indicate that pH alone predicts a majority of the RI distribution in thermal springs (CL, 64%; IPL, 55%), consistent with previous thermal springs studies that concluded pH is the primary predictor of both RI (Pearson et al. 2008; Boyd et al. 2013) and the distribution of genes related to cyclopentyl ring production (Blum et al. 2022). ORP emerges as an important predictor of RI in linear regressions of the YNP dataset (CL = 31%; IPL = 31%), consistent with earlier findings that redox conditions are important for archaeal lipid stress responses (Cobban et al. 2020). Spearman's rho correlations and simple linear regression models highlight the influence of ORP on membrane lipid cyclization. However, ORP has a direct relationship with pH (James et al. 2004), which likely contributes to its association and simple regression significance with RI. Similarly, whilst DO may indicate redox conditions, its temperature‐dependent solubility confounds interpretation of an independent redox state signal. Following ORP (or DO), Fe (II) emerges as a potential control on archaeal membrane GDGT composition (predictive power: CL, 22%; IPL, 22%), but the covariation of Fe (II) with decreasing pH makes it difficult to determine whether Fe (II) exerts any independent effect (Figure S6). Sulphide concentration exhibits minimal predictive power, with influence only on IPL‐RI in a linear regression (11%), but not on CL‐RI, nor any significant Spearman's rho association. As sulphide is a soluble chemical species influenced by pH and redox state, any significant association with iGDGT cyclization may be a result of covariation with other variables. Temperature is associated with CL‐RI but not IPL‐RI, which may be explained by differing CL and IPL crenarchaeol relative abundances in one or two samples that drive the CL correlation over the significance threshold (see Supplement for detailed discussion). It is also possible that temperature influences RI over long time periods (CL), whilst other variables have greater control over ring abundances over shorter time frames (IPL). In other words, IPL composition is more responsive to recent geochemical fluctuations that may not have been captured in this latitudinal study design.

### 4.2 Environmental Predictors of Crenarchaeol Abundance

Crenarchaeol relative abundance, like RI, was associated with multiple environmental variables. Whilst pH, temperature, and redox explain the majority of the variance of RI (MLR: *R* ^2^ = 0.80), temperature and pH are both only weakly associated with crenarchaeol relative abundance (SLR: Temp *R* ^2^ = 0.07, pH *R* ^2^ = 0.03) and combining them adds minimal explanatory power (MLR: *R* ^2^ = 0.11; See Table 1). The variables examined in this study do not sufficiently explain cyclohexyl ring distributions, and simple linear regressions cannot determine the rank order of influence of pH and temperature on crenarchaeol distributions. The relative importance of the two variables also is inconclusive for the non‐linear Spearman's rho tests. The clear tradeoff between statistical power (*N* = 299 and *n* = 41) and geochemical detail (two variables versus seven) between the compiled and the Yellowstone‐only datasets demonstrates the need for consistent variable collection and robust analyses to evaluate a ranked list of variables important for crenarchaeol production.

Whilst temperature is associated with crenarchaeol relative abundance in the global thermal spring dataset, it is not associated with crenarchaeol relative abundance in the more geochemically detailed YNP dataset. The latter observation may be attributed to a lower representation of samples below 60°C for the newly collected YNP dataset (*n* = 2) compared to the global dataset (*n* = 96). This result suggests that temperature is associated with crenarchaeol relative abundance, but that its relationship is weaker than that of pH. Variables present exclusively in our Yellowstone dataset, such as ORP and DO, covary with pH and temperature, and may represent covariation signals. An early examination of crenarchaeol from thermal springs found that bicarbonate concentration (which is partially controlled by the influence of pH on DIC speciation) and not temperature correlated with crenarchaeol distribution (Pearson et al. 2004). Whilst that study measured pH, samples were limited to six regional springs that spanned pH values of 6.4–9.2, a range that would not show the pH signal observed in our current study. A similarly confirmatory study examined 27 YNP hot spring samples and demonstrated that crenarchaeol relative abundance correlated with hot spring chemistry, not temperature (Boyd et al. 2013). The crenarchaeol and crenarchaeol isomer relative abundances in CL fractions correlated with site pH and NO_2_^−^ concentration, whilst CL crenarchaeol relative abundance inversely correlated with NH_4_^+^ concentration. These patterns are consistent with AOA production of crenarchaeol in these springs (Boyd et al. 2013). In IPL fractions, the relative abundances of crenarchaeol correlated with Cl^−^ concentration (indicative of water source) whilst crenarchaeol isomer relative abundance correlated with Fe (II) concentration, which is controlled by pH (Boyd et al. 2013). These results further indicate that hydrothermal water chemistry influences crenarchaeol relative abundance in archaeal lipidomes.

The strong association between pH and crenarchaeol relative abundance is perhaps unsurprising. Without protective adaptations to external pH, a cell's pH homeostasis can be disrupted, thus slowing enzyme activity, destabilising other proteins and nucleic acids, and potentially resulting in cell death (Slonczewski et al. 2009). As most literature on iGDGT distributions is focused on marine paleoclimate applications, the influence of pH on crenarchaeol abundance has remained uncharacterized due to the narrow range of pH observed in the marine realm. The average pH of Earths' surface oceans is 8.07 ± 0.02 between 60° North and 60° South (Jiang et al. 2019), but *Nitrososphaeria* also thrive in less alkaline marine thermoclines (pH 7.5–7.2 from 200 to 600 m depth; Clayton and Byrne 1993; Palmer et al. 1998; Church et al. 2010). The wide pH range of terrestrial thermal springs (1.14–10.10) covered in this compilation shows that crenarchaeol is present over a broad range of pH values (1.96–10.1) but is most abundant (> 30% of core/total iGDGTs) from pH 6.0 to 9.2. Whilst a pH range from 6.0 to 9.2 is relatively narrow for thermal springs, it is much wider than that of the modern surface ocean (8.0–8.25; Jiang et al. 2019).

Differences in parameters other than pH that contribute to multiple linear regression models of crenarchaeol and RI may indicate that nuanced or independent processes influence production of cyclohexyl rings in iGDGTs or may reflect co‐variation of soluble chemical parameters with pH and temperature. In addition to pH, DO and Fe (II) are statistically important predictors of RI, whilst those of crenarchaeol relative abundance are SPC, ORP, and temperature. Whilst these secondary variables could cause membrane stress that would alter lipid production (Cobban et al. 2020; Zhou et al. 2020), it may be more likely that these weak correlations of soluble geochemical parameters with lipid compositions are due to co‐variation with parameters such as pH and temperature. Whilst cyclohexyl rings could have a specific relationship to cellular redox conditions, this is not supported by culture experiments that have altered redox state and observed no significant change in crenarchaeol production (Qin et al. 2015).

### 4.3 Temperature and pH Optima for Crenarchaeol Production

**FIGURE 5:**
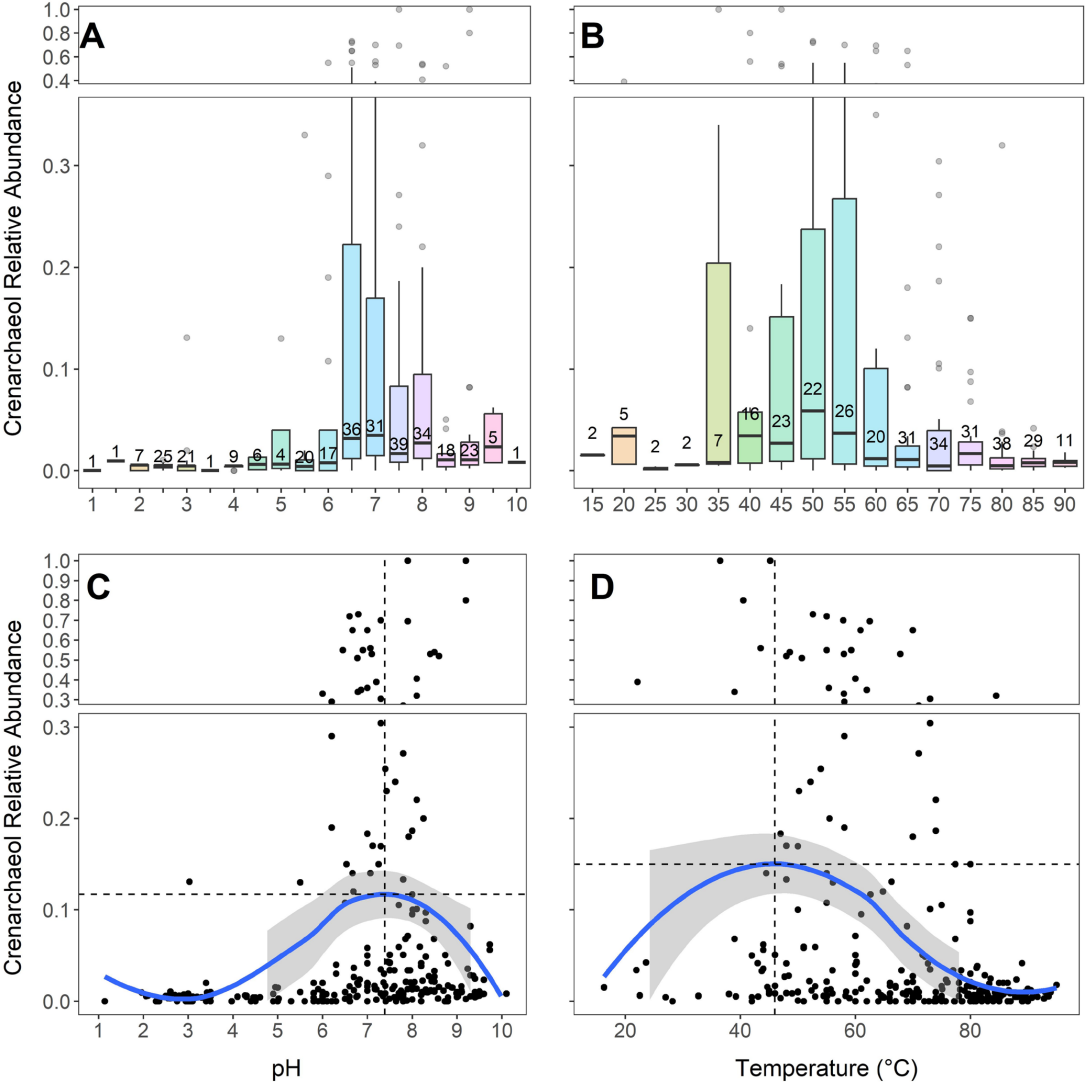
Boxplots (A, B) and LOESS smoothing curves (C, D) of crenarchaeol relative abundances from all compiled data (*N* = 299). Data ranges are binned by 0.5 pH units (A) and 5°C temperature units (B) with bins labelled by their minimum bound. Median and quartile abundances are visualised in boxes and maximum and minimum values are indicated by whiskers. Sample counts for each bin are included above mean value indicators in A and B. The functions plotted in C and D have 95% confidence intervals shaded in grey. Dashed lines in C and D represent *x* and *y* values where crenarchaeol reaches its maximum. *Y*‐axis breaks and scale changes are utilised to allow outliers to be visualised.

To test the hypothesis that crenarchaeol may optimise (hyper)thermophilic membranes for more mesophilic settings, we estimate the optimal temperature and pH for crenarchaeol production using our global compilation (total data, *N* = 299; Figure 5). The pH range (discussed hereafter as bins shown in Figure 5) with the highest mean crenarchaeol relative abundance is 7.0–7.5 whilst bins from pH values of 6.5–8.5 also have high sample counts with elevated means and upper quartiles relative to those outside of this pH range (Figure 5A). The bin from pH 9.5–10 also has high crenarchaeol relative abundance (0.01–0.06), but this may be an artefact of the small site number (*n* = 5), demonstrating the need for further quantification of archaeal lipids in alkaline springs. The temperature bin with the highest mean crenarchaeol relative abundance spans 50°C–55°C, whilst bins from 40°C to 60°C have elevated mean and upper quartile values relative to those outside this range (Figure 5B). In general, bins from 40°C to 90°C have high sample counts whilst bins from 15°C to 40°C and above 90°C have low sample counts, explaining the high upper quartile value of the bin from 35°C to 40°C (*n* = 7).

The LOESS (locally weighted smoothing; Figure 5C,D) functions show the optimal environmental pH for maximising the relative abundance of crenarchaeol is 7.4 (corresponding to 0.12 LOESS‐predicted relative abundance), consistent with the boxplot having the highest mean (pH 7–7.5) in Panel 5A. The optimal temperature for maximising the relative abundance of crenarchaeol is 46°C (0.15 LOESS‐predicted relative abundance). Whilst this value is not within the highest‐mean bin of Panel 5B (50°C–55°C), it does fall within the broad grouping of temperatures showing elevated mean crenarchaeol relative abundances (40°C–60°C); this is consistent with temperature having a weaker overall control on iGDGT, and specifically crenarchaeol, patterns compared to pH. Scatter in Panel 5D shows that crenarchaeol can also be found in high relative abundances above 80°C. Note that thermal spring data are expected to have some degree of noise due to environmental variability over several timescales including changes in volcanic/hydrothermal activity, seasonality, and diurnal cycles (Payne et al. 2019; Colman et al. 2024; see Supplement for further discussion).

The analysis shown in Figure 5 corroborates the observed circumneutral pH threshold identified in Figure 3, in which instances of abundant crenarchaeol track the temperature‐dependent neutral pH line. The directionality of the relationship of crenarchaeol with environmental parameters is consistent with previous hypotheses that crenarchaeol acts to expand archaeal membranes (in opposition to cyclopentyl rings; Sinninghe Damsté et al. 2002). Acidic environments have an excess of protons, promoting the need for cell membrane compaction through cyclopentyl ring production to prevent energetic imbalances (Boyd et al. 2011). If crenarchaeol expands membrane fluidity, crenarchaeol concentrations should be expected to be lower in acidic environments. Whilst there exists an abrupt acidity cutoff for high relative crenarchaeol production, there is no apparent upper pH limit (Figure 3). This may indicate that high pH is not as stressful as acidic pH for crenarchaeol‐producing archaeal cells. Nevertheless, further study of alkaline environments from pH 9 to 12 could illuminate a decrease in crenarchaeol production if an excess of hydroxyl ions poses significant stress to archaeal cells. Another consideration when interpreting the pH optimum of crenarchaeol is the pH‐dependent speciation of soluble metabolic substrates, such as NH_3_ utilised during nitrification by AOA. Whilst the p*K*_a_ of the NH_4_^+^‐NH_3_ system is strongly temperature dependent (Amend and Shock 2001), NH_3_ is scarce in acidic settings, potentially decreasing crenarchaeol production by AOA that lack metabolic substrates in acidic systems. However, the production of crenarchaeol in environments in which AOA are rare (> 75°C, acidic pH) warrants further explanation, as discussed below in Section 4.4.

### 4.4 Explanations for Crenarchaeol Optima in Natural Hydrothermal Springs

#### 4.4.1 Environmental Distributions of Crenarchaeol‐Producing Archaea

A parsimonious interpretation of the crenarchaeol optima estimated from our environmental data may be that the organisms producing crenarchaeol grow and synthesise the lipid optimally in circumneutral and mesophilic settings. This interpretation is consistent with cultivation studies that demonstrate that AOA from the class *Nitrososphaeria* are most abundant below ~75°C and at or above neutral pH (Könneke et al. 2005; De La Torre et al. 2008; Hatzenpichler et al. 2008; Elling et al. 2015, 2017). The correlation of *amoA* gene abundance with the distribution of crenarchaeol in 27 thermal spring samples (Boyd et al. 2013) similarly supports optimal crenarchaeol production in circumneutral and mesophilic springs. However, AOA are absent or scarce in hyperthermal (> 75°C) springs (Hou et al. 2013; Podar et al. 2020; Colman et al. 2024), indicating that other archaeal taxa may be producing crenarchaeol at higher temperatures and potentially at moderate spring conditions. Not all archaea in the class *Nitrososphaeria* are AOA, and novel groups have been shown to be thermoacidophilic (Beam et al. 2014; Kato et al. 2019). The non‐AOA group *Conexivisphaerales* is abundant in YNP hydrothermal springs below pH 5 (Figure S2; analysis of the *Nitrososphaeria* metagenome‐assembled genome (MAG) data from Colman et al. 2024). The AOA groups demonstrate the expected preference for circumneutral to alkaline settings, whilst the non‐AOA groups inhabit either acidic or circumneutral pH ranges (Colman et al. 2024; Figure S2). So far, one thermoacidophilic isolate exists from the non‐AOA *Nitrososphaeria* (*Conexivisphaera calidus*; Kato et al. 2019), but its lipidome remains uncharacterized. Therefore, it remains unknown whether the acidophilic *Nitrososphaeria* produce crenarchaeol, but the presence of only trace amounts of crenarchaeol below pH 5 indicates that crenarchaeol does not constitute a large portion of the membrane in *Conexivisphaerales* or other *Nitrososphaeria* from lower pH systems. In contrast, a non‐AOA *Nitrososphaeria* group, the *Caldarchaeales*, preferentially inhabit circumneutral settings and are present in hyperthermal springs (Colman et al. 2024; Figure S2). Whilst a tungsten‐dependent representative of the *Caldarchaeales* (*Wolframiiraptor gerlachensis*) has been cultivated in a stable enrichment culture (Buessecker et al. 2022), the lipidomes and potential for crenarchaeol production of *Caldarchaeales* members remain uncharacterized. Future work to characterise the membrane composition of non‐AOA *Nitrososphaeria* isolates and to identify the gene(s) encoding the enzyme(s) for cyclohexyl ring formation will be critical to constrain the phylogenetic distribution of crenarchaeol production and will allow us to better differentiate phylogenetic versus geochemical controls on crenarchaeol distributions in nature and throughout the geologic record. Similarly, testing for correlations between crenarchaeol abundance and different taxonomic groups could either support or contradict the long‐standing prediction that crenarchaeol is specific to *Nitrososphaeria* (Pearson and Ingalls 2013; Schouten et al. 2013). Laboratory experiments aiming to produce abundant crenarchaeol from isolate cultures may benefit from targeting our predicted optimal temperatures from 40°C to 55°C and pH values from 6.5 to 8.0 (Figure 5A,B), whilst incorporating comparative transcriptomics and proteomics may help identify putative cyclohexyl ring biosynthesis enzyme(s).

#### 4.4.2 Crenarchaeol Membrane Dynamics

The circumneutral and mesophilic crenarchaeol optima estimated in this study may also be interpreted to be consistent with the hypothesised function of crenarchaeol to decrease membrane lipid packing. AOA are thought to have radiated from a thermophilic ancestor (Abby et al. 2020; Luo et al. 2024), which would have produced lipid membranes with high cyclopentyl ring abundances to protect against extremes in temperature. Upon expansion into cooler marine waters, these organisms would have required a mechanism to increase membrane fluidity to adapt to less ‘stressful’ environments. Whilst our data cannot determine whether crenarchaeol facilitated this evolutionary transition, our environmental optima and the thermophilic nature of AOA precursors agree with the hypothesised membrane‐expanding function of crenarchaeol. Our analysis spans a larger range of environmental variables and has a higher sampling density than previous studies, indicating that the temperature optimum of crenarchaeol from this study (46°C) is likely representative, whilst the prior estimated optimum of 40°C (Zhang et al. 2006) is within the elevated range of the 95% confidence interval of the model (Figure 5). Our temperature optimum agrees with the respective 30°C–65°C (Robert and Chaussidon 2006) and 47°C (Grossman and Joachimski 2022) sea‐surface temperature reconstructions during the predicted dates of the transition of AOA into the ocean (1017 Ma, Ren et al. 2019; 509 Ma, Yang et al. 2021) and the 33°C–42°C range (Bice et al. 2006) during the proposed expansion of non‐thermophilic marine *Nitrososphaeria* during the mid‐Cretaceous anoxic event (ca. 112 Ma; Kuypers et al. 2001). However, recent molecular dynamics simulations suggest that the cyclohexyl ring decreases membrane fluidity (Zhou and Dong 2025), which is consistent with cyclopentyl ring effects that indicate high‐temperature adaptation. This contrasts with the membrane‐expanding function indicated by previous molecular studies on crenarchaeol and our environmental analysis. Additionally, the proportion of crenarchaeol in the membrane of a cultured thermophilic AOA (*Nitrosotenius uzonensis*) increased with increased growth temperature from 37°C to 46°C to 50°C (Bale et al. 2019), which was attributed to the fluidity‐decreasing function of crenarchaeol. These results are not inconsistent with a mesophilic optimal temperature of crenarchaeol production, as the maximum temperature examined here is within our estimated optimal temperature range. Interestingly, the molecular dynamics simulations also suggest that the cyclohexyl ring increases membrane permeability (Zhou and Dong 2025), which contrasts with cyclopentyl ring effects and indicates that parameters other than temperature may drive crenarchaeol expression. Our finding that crenarchaeol is most closely related to pH suggests that the cyclohexyl rings may be significant in maintaining a proton gradient across the cellular membrane through permeability modifications (Chong 2024; Zhou and Dong 2025). Therefore, crenarchaeol may have a nuanced function compared to other iGDGTs in which pH and other permeability‐dependent conditions drive its production, rather than temperature. Future studies examining the distribution of crenarchaeol in the geologic record and identifying the gene(s) encoding the enzyme(s) for cyclohexyl ring formation will allow determination of the potential function of crenarchaeol in the evolutionary history of archaea. The current study provides the environmental context for mechanistic investigations into the distribution of crenarchaeol in geochemically diverse settings.

## Author Contributions

**Amanda N. Calhoun:** formal analysis, project administration, data curation, writing – review and editing, visualization, validation, methodology, writing – original draft, investigation, conceptualization, funding acquisition, software, resources. **Jerome Blewett:** investigation, methodology, writing – review and editing, project administration, supervision, resources. **Daniel R. Colman:** writing – review and editing, resources, supervision, project administration, visualization, methodology, funding acquisition, data curation. **Maximiliano J. Amenabar:** resources, writing – review and editing, methodology. **Carolynn M. Harris:** writing – review and editing, methodology, resources. **Eric S. Boyd:** writing – review and editing, methodology, supervision, resources, project administration, funding acquisition, conceptualization. **Ann Pearson:** writing – original draft, funding acquisition, resources, supervision, data curation, project administration, visualization, writing – review and editing, methodology, conceptualization, investigation. **William D. Leavitt:** conceptualization, investigation, funding acquisition, writing – original draft, writing – review and editing, visualization, project administration, supervision, resources.

## Funding

This work was supported by the National Science Foundation (1928309), National Aeronautics and Space Administration (80NSSC19M0150), American Chemical Society Petroleum Research Fund (66614‐ND2), and Stamps Foundation.

## Conflicts of Interest

All authors have contributed substantially to the work and approved the final version of the manuscript. The authors declare no conflicts of interest, and authors Eric S. Boyd and Daniel R. Colman are not involved in the editorial process. All local, national, and international regulations and conventions, in addition to normal scientific ethical practises, have been respected. We provide consent for publication in EM, if accepted.

## Supporting information


**Data S1:** Supplementary Information.

## Data Availability

The work presented here is original research and has not been submitted for publication elsewhere. A pre‐print of the manuscript has been posted to the bioRxiv server, and all data will be permanently archived in FigShare whilst code is made available in GitHub (https://zenodo.org/doi/10.5281/zenodo.12603497).
