# Supplementary material for: Environmental Controls on Crenarchaeol Distributions in Hydrothermal Springs
Source: Environ Microbiol. 2026 Feb 16;28(2):e70248. doi: 10.1111/1462-2920.70248 (PMC12909067; doi:10.1111/1462-2920.70248)
Supplement: Supplementary file 1 — Data S1: Supplementary Information. [file EMI-28-e70248-s001.docx]

# **Supplemental Information**

# **Figures**

**
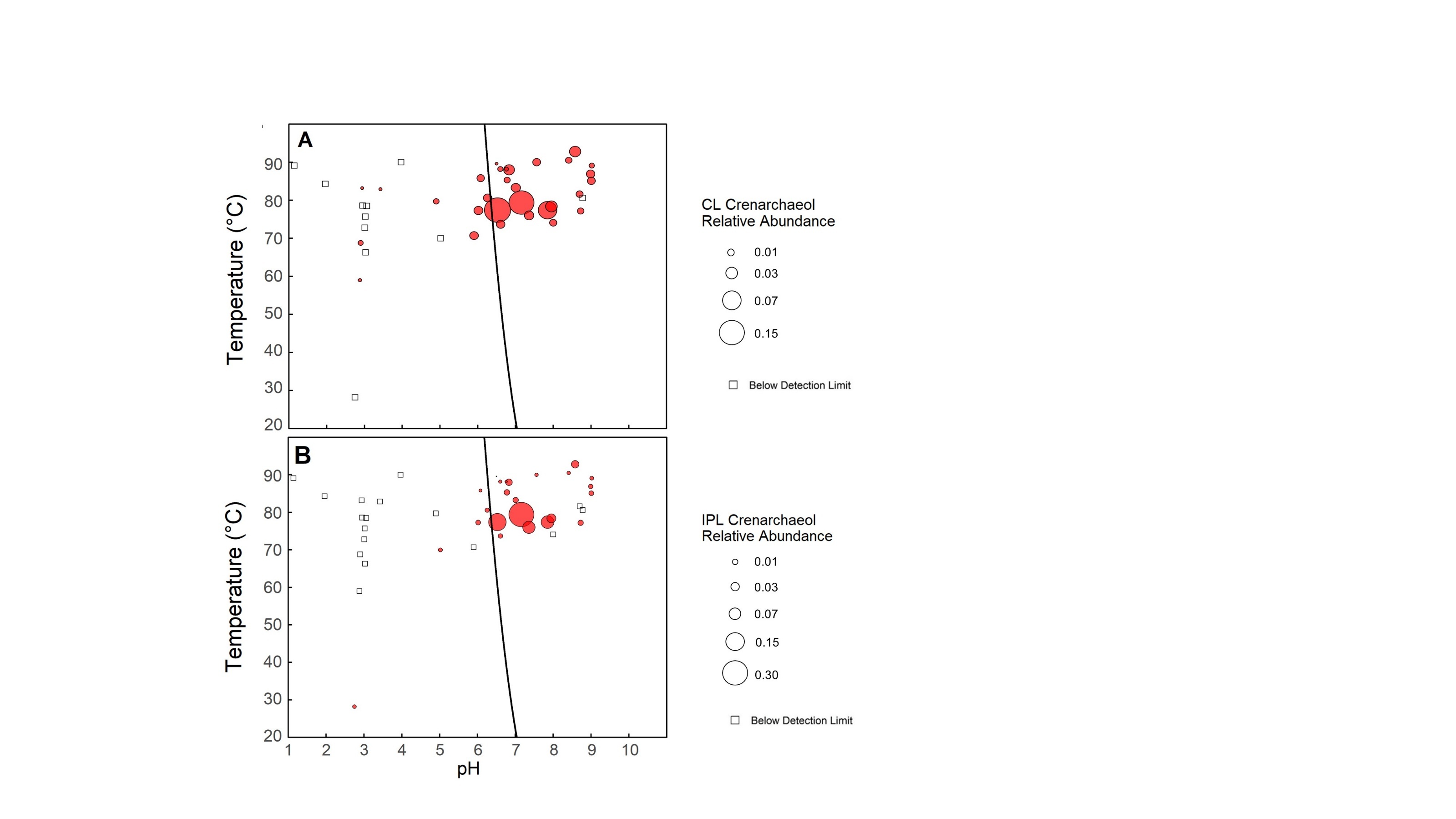
**

**Figure S1.** Relative abundances of (A) CL and (B) IPL crenarchaeol in temperature-pH space for 41 Yellowstone field samples collected from 2018-2022. Bubble area is proportional to relative abundance of crenarchaeol while squares represent non-detectable abundances. The black curve is the temperature-dependent neutral pH line (Equation 3).

**
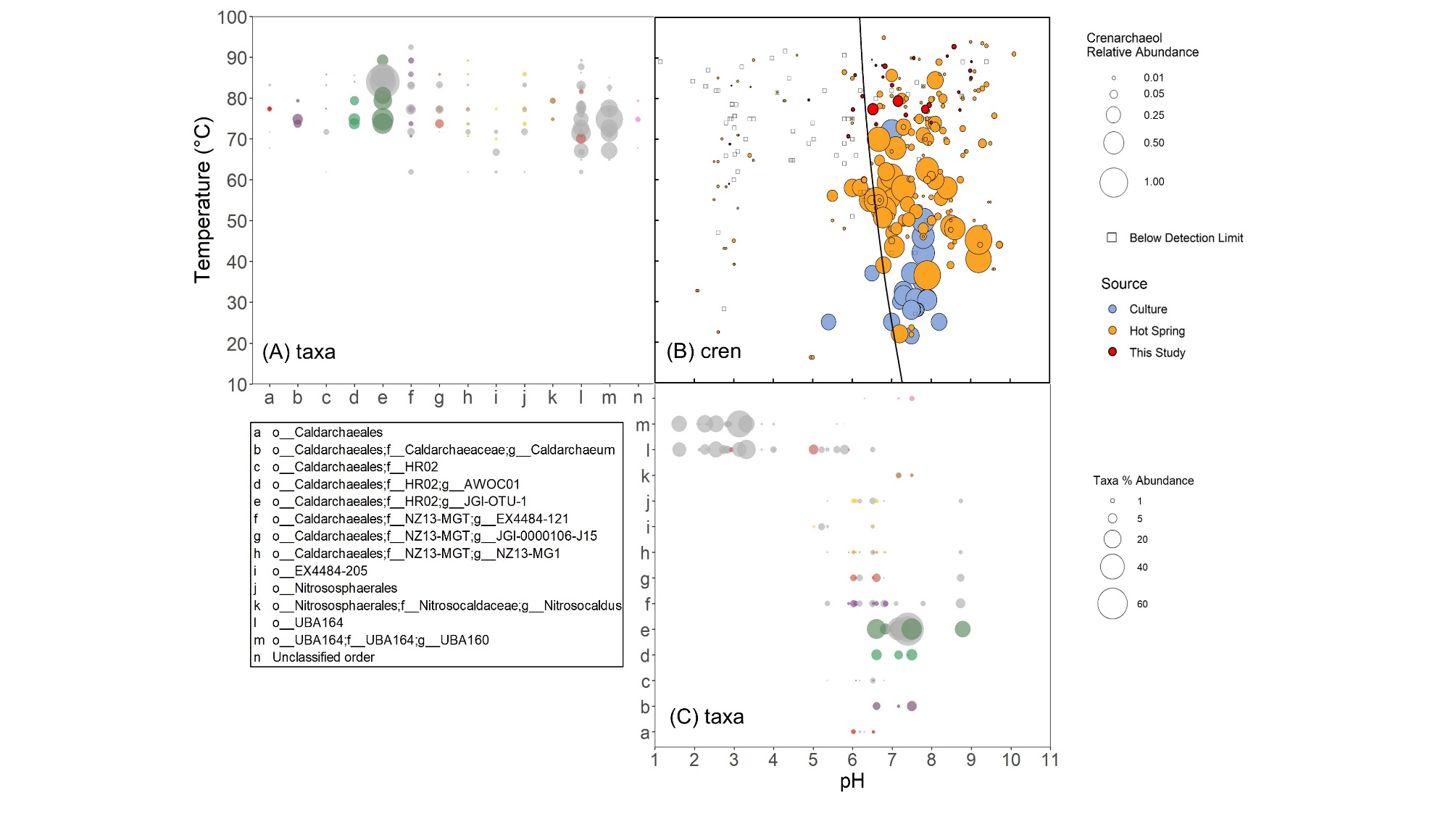
**

**Figure S2.** Taxa percent abundances of *Nitrososphaeria* groups identified in Yellowstone thermal springs (data from Colman et al., 2024) across temperature (A) and pH (C) gradients compared to crenarchaeol relative abundance (B) from main text Figure 3. Data are from 1022 metagenome-assembled genomes (MAGs) of 34 high-temperature, chemosynthetic springs in Yellowstone National Park and 444 MAGs from 35 published metagenomes. Taxa percent abundance is relative to all taxa, not just *Nitrososphaeria* groups. Points in color in panels (A) and (C) correspond to 17 springs that overlap with sites the current study examined for crenarchaeol abundance, while points in gray are from 17 sites from Colman et al. (2024) for which we do not have lipid abundance data.

**
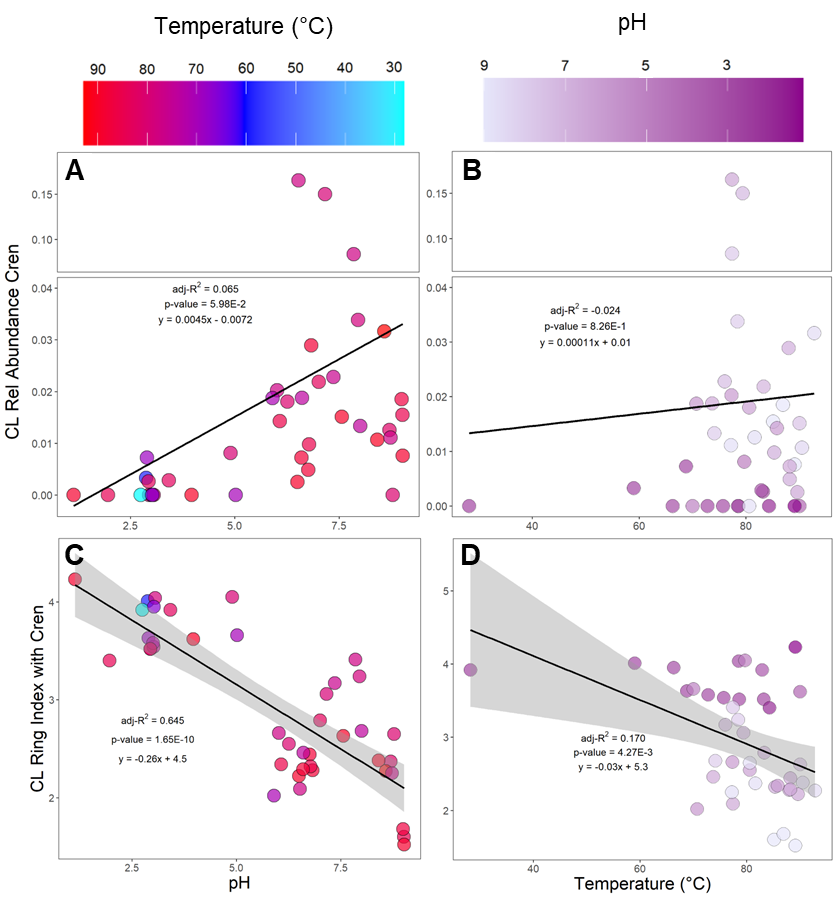
Figure S3.** Relative abundances of CL crenarchaeol versus (A) pH and (B) temperature and Ring Index (with cren) versus (C) pH and (D) temperature for Yellowstone samples (n = 41) with fill by the other variable. Equations of lines of best fit, p-values, and adjusted-R^2^ values are included. A y-axis break and scale change are utilized to include high abundance outliers of crenarchaeol abundance while visualizing detailed distributions of the majority of samples. Significant RI linear relationships have 95% confidence intervals shaded in gray.


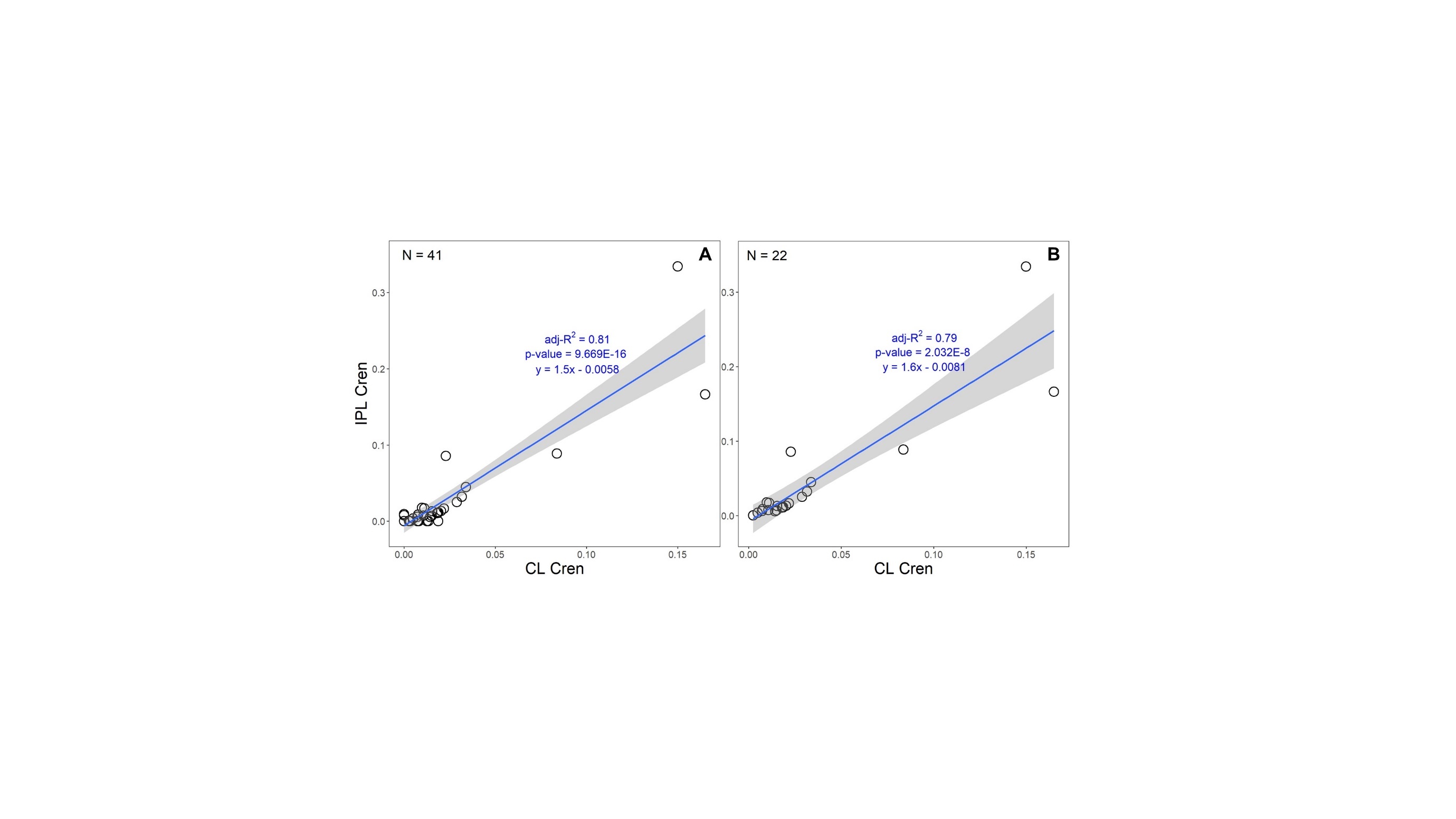


**Figure S4.** Linear regression model of IPL versus CL crenarchaeol relative abundance for each Yellowstone site. Panel (A) includes all sites while panel (B) excludes sites with zero values for CL or IPL crenarchaeol. The 95% confidence interval is shaded in gray and summary statistics are included.


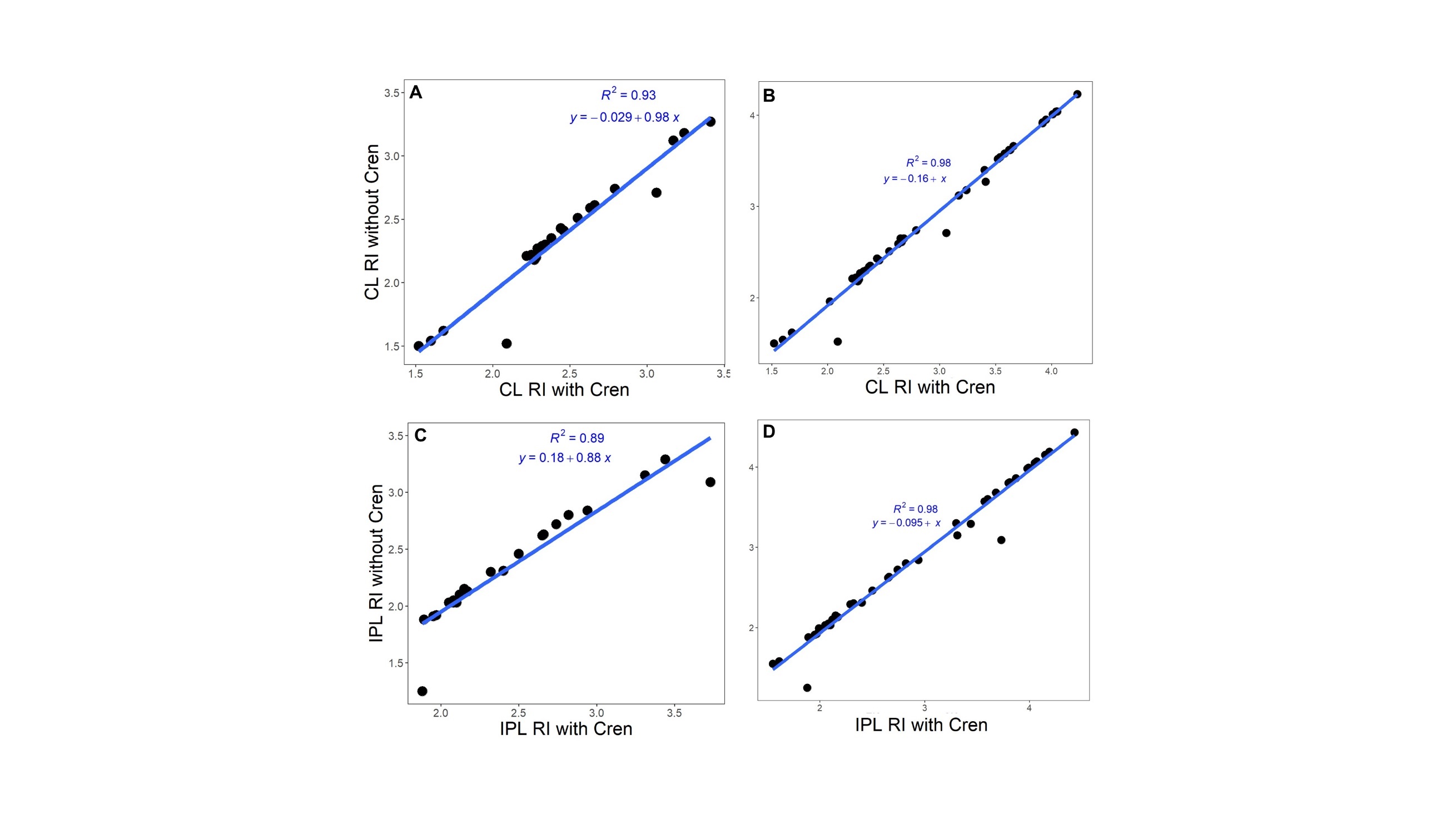


**Figure S5.** Simple linear regressions between Ring Index calculated without and with crenarchaeol for CL and IPL GDGT fractions from Yellowstone samples (See Equations 1 and 2). Panels (A) and (B) represent thermal springs with detectable amounts of crenarchaeol (n = 30), while Panels (C) and (D) have values from all 41 thermal spring samples regardless of crenarchaeol presence.


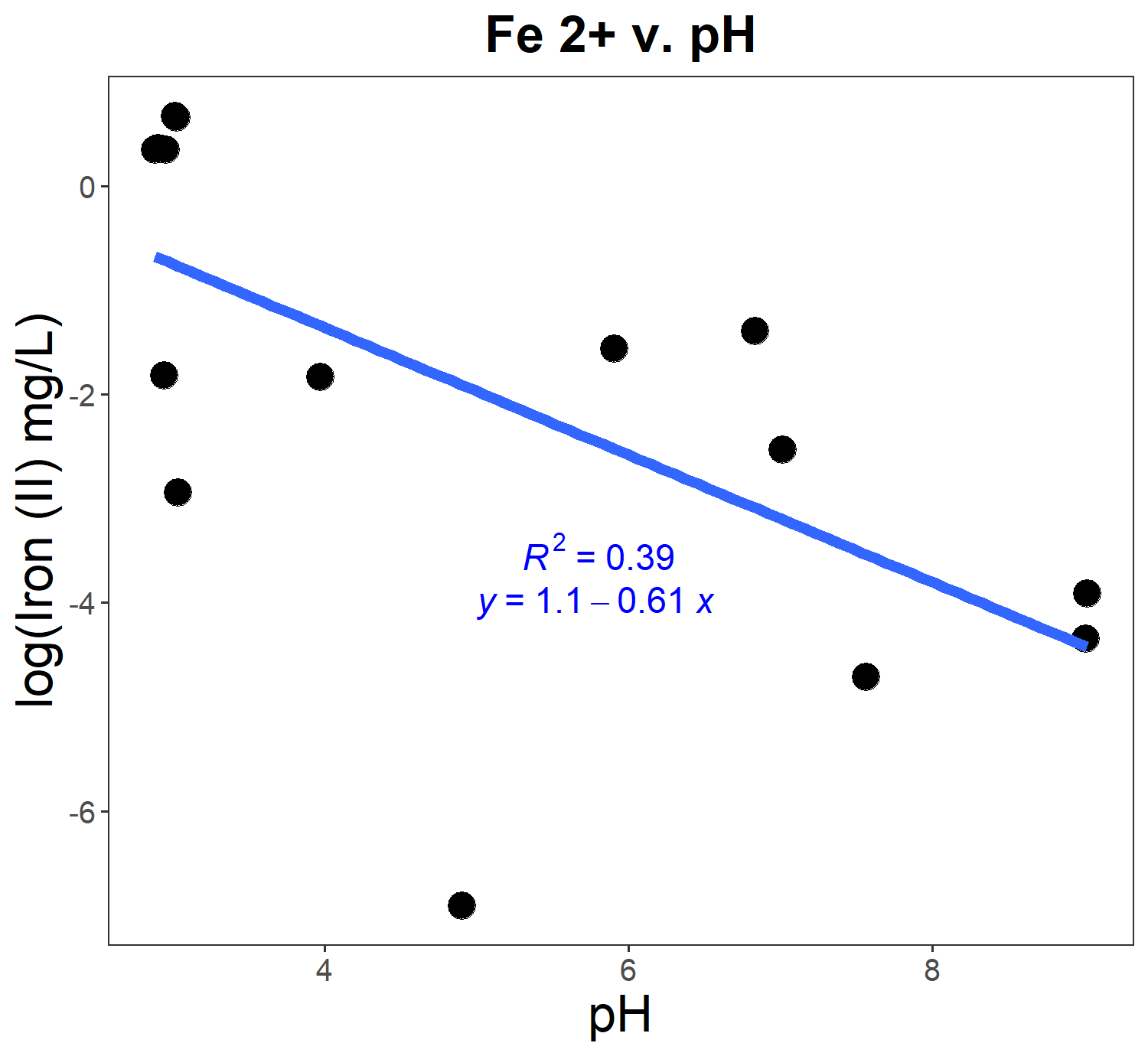
**Figure S6.** Simple linear regression of the log of Fe (II) concentration with pH for thermal springs with data for both parameters (n = 31). Non-finite log values are excluded, and the Spearman’s rho correlation p-value is 9.73 x 10^-5^.

# **Supplemental Tables**

## **Yellowstone Crenarchaeol**

**Table S1.** Field geochemical and geophysical parameters, crenarchaeol relative abundances, and ring indices for 41 samples from Yellowstone National Park collected during field seasons from 2018-2022.


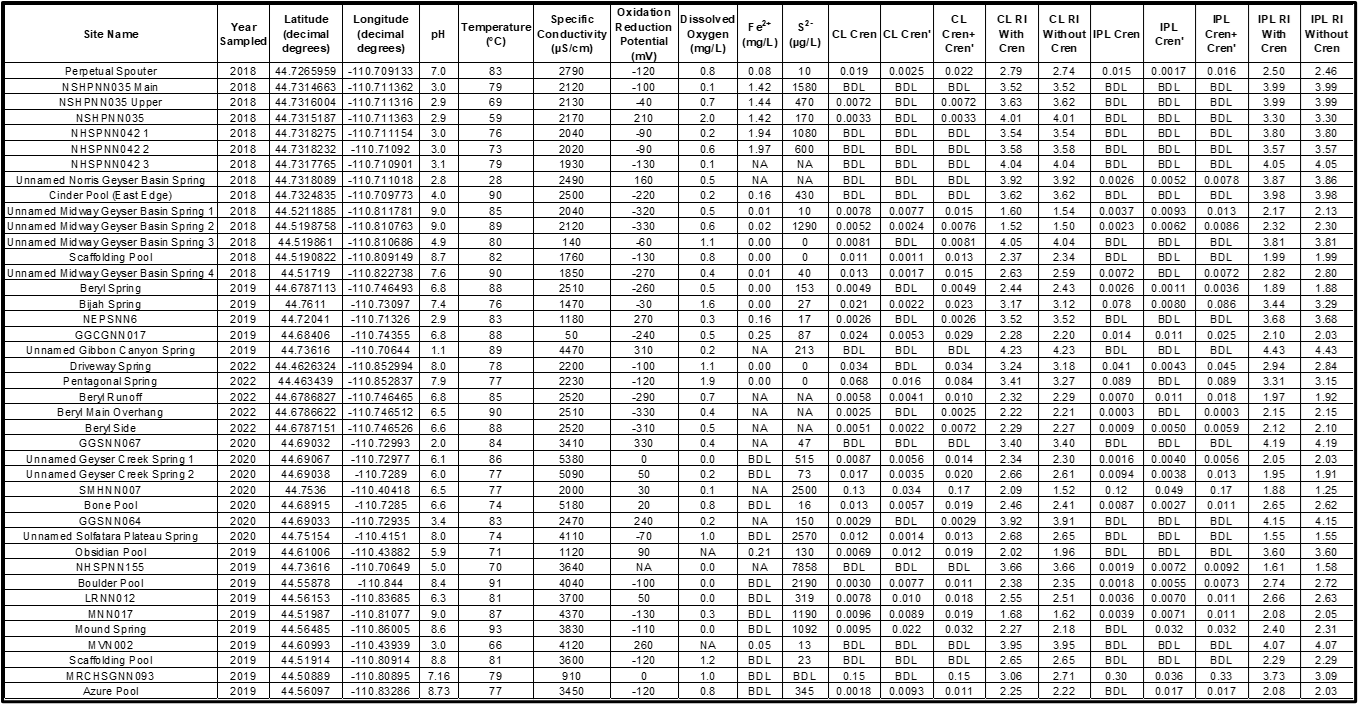


**Table S2** Spearman’s rho correlation p-values for environmental parameters and crenarchaeol relative abundance from Yellowstone samples. Analyses are run for both core (CL) and intact polar lipid (IPL) fractions and significant correlations are indicated in bold italics.


**Table S3**: Outputs of simple linear regression models for environmental parameters and CL and IPL relative abundance of crenarchaeol from Yellowstone samples. The significance threshold is set to a p‑value of 0.05 and any significant correlations would be indicated in bold italics.

**Table S4.** Outputs of 10 multiple linear regression models with highest adjusted R^2^ values for environmental parameters and relative abundances of CL crenarchaeol from Yellowstone samples. The significance threshold is set to 0.05 and significant p‑values are indicated in bold italics. Models are reported in order of decreasing adjusted R^2^ values.

## **Yellowstone Ring Index**

**Table S5.** Spearman’s rho correlation p-values for environmental parameters and Ring Index from Yellowstone samples. Analyses are run for both core (CL) and intact polar lipid (IPL) fractions and significant correlations are indicated in bold italics.

**Table S6.** Outputs of simple linear regression models for environmental parameters and CL and IPL Ring Index with crenarchaeol from Yellowstone samples. The significance threshold is set to a p‑value of 0.05 and significant correlations are indicated in bold italics.

**Table S7.** Outputs of 10 multiple linear regression models with highest adjusted R^2^ values for environmental parameters and CL Ring Indices with crenarchaeol from Yellowstone samples. The significance threshold is set to 0.05 and significant p‑values are indicated in bold italics. Models are listed in order of decreasing adjusted R^2^ values.

# Supplementary Information

## **Comparisons of Core and Polar Fractions**

To identify whether studies should split GDGTs into CL and IPL fractions, we compared our YNP results for these two fractions. CL and IPL crenarchaeol relative abundances are strongly positively correlated (R^2^ = 0.81; Figure S4). Interestingly, the slope of this relationship is 1.5, indicating that on average, crenarchaeol has greater relative abundance in IPL fractions than in CL fractions. IPL lipids are thought to derive from extant microbial communities and CL lipids record long-term production, indicating that microbial communities in sampled YNP springs may have been producing more crenarchaeol upon collection than the time integral represented in the CL fraction (Lipp and Hinrichs, 2009). Another hypothesis is that crenarchaeol is more labile than the other GDGTs and its CL abundance decreases over time relative to other GDGTs. To distinguish between these two hypotheses, we plotted IPL Ring Index with crenarchaeol versus CL Ring Index with crenarchaeol, which produced a linear trendline with the equation y = 0.88x + 0.35 (R^2^ = 0.58). The result of plotting IPL RI without cren versus CL RI without cren is almost identical with a trendline of y = 0.88x + 0.34 (R^2^ = 0.60). The slopes of 0.88 are close to a 1:1 relationship, different from the crenarchaeol abundance slope of 1.5 for IPL versus CL. This indicates that crenarchaeol may be preferentially degraded compared to other GDGTs with just cyclopentyl rings, supporting the degradation hypothesis over the environmental change hypothesis.

Four sites deviate from the best-fit line and lie outside of the 95% confidence interval in Figure S4, but these outliers maintain a positive relationship between CL and IPL crenarchaeol. The four samples all have relatively high CL and IPL crenarchaeol relative abundances for the Yellowstone dataset. Two sites, SMHNN007 (CL = 0.17; IPL = 0.17) and Pentagonal Spring (CL = 0.08; IPL = 0.09) have more CL crenarchaeol than predicted by the best-fit line with a slope of 1.5. Both springs have similar relative abundances of crenarchaeol in CL and IPL fractions, indicating the native microbial cells were producing their time-averaged proportion of crenarchaeol at the time of sampling from potentially stable geochemical conditions. Another possibility is that IPL crenarchaeol degradation is enhanced in these springs.

The other two high relative abundance sites are MRCHSGNN093 (CL = 0.15; IPL = 0.33) and Bijah Spring (CL = 0.02; IPL = 0.09), which have more IPL crenarchaeol than predicted by the best fit line. The greater relative abundance of IPL crenarchaeol indicates that either the degradation rate of IPL crenarchaeol is lower in these springs, or that the microbial community at the time of sampling of these springs was producing more crenarchaeol relative to other GDGTs than the historical average of this spring. This phenomenon may indicate geochemical conditions that inhibit IPL crenarchaeol degradation, seasonal variation in environmental parameters that influence crenarchaeol relative abundance, or recent shifts in microbial community or geochemical/geophysical conditions of the springs. Hydrothermal conditions of springs are known to vary with major geyser eruptions, hydrologic changes, earthquakes, discharge rates, variable mixing of gases/waters, and annual and seasonal disturbances, which could explain these differences in CL and IPL crenarchaeol (Heasler and Jaworowski, 2018).

Conditions experienced by microorganisms in thermal spring environments are also known to be influenced by the volcanic system that drives hydrothermal activity in Yellowstone National Park, which undergoes complex fluctuations over time (Hurwitz and Lowenstern, 2014). Climate variation may also play a role in altering thermal spring temperature and chemistry, which would influence trends observed for the 41 Yellowstone samples collected during different times of the year. Variations over even smaller temporal scales can influence thermal spring conditions. Samples were collected from thermal springs across Norris Geyser Basin, which is known to have ground temperature variation from ~30°C to ~50°C over the course of a single day (Neale et al., 2016). Long-term increases in annual mean air temperature may represent more systemic changes for spring chemistry through indirect processes such as precipitation and nutrient flux, while drought conditions alter input from water sources such as volcanically heated reservoirs, riverine input, and precipitation (Heeter et al., 2021). During the 2022 field season, sediment samples were collected shortly after significant snowfall, which may have influenced spring geochemistry both by direct input and alteration of water table level. Differences in source-water depths of these hydrothermal systems also have important implications for the geochemistry of these springs (Fournier et al., 2002).

The variable conditions of thermal springs in Yellowstone may explain discrepancies between crenarchaeol presence/absence in CL and IPL fractions from the same sample. Out of 41 samples generated in this study, eight have detectable levels of CL crenarchaeol and no detectable levels of IPL crenarchaeol (see Excel file of data table). Interestingly, two have detectable levels of IPL crenarchaeol and no detectable levels of CL crenarchaeol. Samples with measurable amounts of CL crenarchaeol and non-detectable IPL crenarchaeol are expected and can be explained by the cessation of crenarchaeol production in these springs with environmental variation or microbial community change because core lipids are degradation products of intact polar lipids. In contrast, the presence of detectable amounts of IPL crenarchaeol and no CL crenarchaeol in two sites is more difficult to explain. One of these sites is a temperature outlier, Unnamed Norris Geyser Basin Spring (28.2°C, pH 2.75), which may have recently begun crenarchaeol production and has a slow degradation rate of archaeal lipids at the relatively low temperature of this spring. The other anomaly, NHSPNN155 (70°C, pH 5.02), does not support this slow-degradation interpretation. In NHSPNN155, the only anomaly is the high dissolved sulfide level, at 2.45 x 10^-4^ M. This is the highest value of the Yellowstone dataset, with a second-highest concentration being 8.01 x 10^-5^ M. A majority (75%) of samples with S^2-^ data available (36 sites) are at least an order of magnitude lower with an average concentration of 700 µg L^-1^. Upon removal of NHSPNN155 from this dataset average, the value drops to 496 µg L^-1^. Sulfide concentration was not measured for Unnamed Norris Geyser Basin Spring, which prevents comparison of sulfur abundance across these two sites. However, lipid sulfurization is a known diagenetic process that may lead to enhanced degradation of CL GDGTs in the presence of high sulfur concentrations, which could lead to non-detectable levels of CL crenarchaeol (Schouten et al., 2013; Adam et al., 2000). While there are exceptions, the majority of samples (75.6%) have consistent crenarchaeol presence and absence across CL and IPL fractions.

The strong linear relationship between CL and IPL crenarchaeol relative abundances justifies plotting results of only CL fraction analyses because similar statistical results are observed for IPL fractions. Multiple linear regression analyses were also run for IPL crenarchaeol to confirm similarity of results with those for CL crenarchaeol reported in Table S3. While results should do not vary between fractions, we chose to plot results for CL crenarchaeol rather than IPL crenarchaeol because CL lipids record a longer history of GDGT production that is not as subject to recent seasonal changes as expected for IPL crenarchaeol (Lipp and Hinrichs, 2009). In addition, we utilized internal GTGT standard to calculate rough absolute abundances of GDGTs in the samples. CL fractions represent the majority of the GDGTs from 94% of samples with internal standard (n = 33), making them preferable for robust quantification on the QQQ and use in analyses. Two samples, Unnamed Midway Geyser Basin Spring 2 and Driveway Spring had more IPL than CL GDGTs (57% and 84%, respectively), which is anomalous in this dataset. The other 31 samples whose absolute abundance could be calculated had an average percent CL composition of 85.9% ± 13.0, establishing a significant dominance of CL lipids in these samples. The rough calculations from addition of GTGT standard are imprecise and are best suited for order-of-magnitude calculations, which prevents accurate combinations of absolute GDGT abundances from IPL and CL data. Past studies have varied in the lumping or splitting of CL and IPL fractions, so we report compiled literature samples as either total isoprenoid-GDGT abundances or CL abundances (Boyd et al., 2013; Xie et al., 2015). In comparing the literature values to the new data generated in this study, we present only CL crenarchaeol data due to the higher magnitude of CL versus IPL lipid absolute abundances and representation of a long record of GDGT production.

While differences between CL and IPL crenarchaeol relative abundances can indicate recent shifts in microbial community or environmental conditions in each spring, broad trends of cyclopentyl ring abundances (represented by RI) are consistent across CL and IPL fractions (see Table S4 and Table S5). Given this observation, it may be sufficient to analyze relative GDGT abundances amongst total isoprenoid-GDGTs, rather than splitting into CL and IPL fractions, depending on the scientific goal. This is significant because splitting samples and processing fractions in tandem involves labor intensive wet chemistry protocols and doubles analytical machine time. However, if the environment in question has undergone significant recent changes or researchers are interested in a lipid of high abundance, fraction splitting is suggested to obtain a comprehensive view of lipid production.

## **Effect of Crenarchaeol on Ring Index Calculations**

We compared YNP Ring Index calculated with and without crenarchaeol in Supplemental Figure S5 and demonstrate a strong 1:1 correlation between the two, justifying our utilization of RI calculated with crenarchaeol subsequent analyses. Panels A and C show regressions of YNP samples with detectable crenarchaeol for CL (R^2^ = 0.93) and IPL fractions (R^2^ = 0.89), indicating a strong correlation of RI values. The slopes of the significant CL and IPL correlations are 0.98 and 0.88, respectively, which are close to the 1:1 relationship expected for RI values from the same sample. Two samples lie outside of the 95% confidence interval of the regression relationships, which are from MRCHSGNN093 and SMHNN007, the springs with the highest relative abundances of both CL and IPL crenarchaeol in the Yellowstone dataset (CL = 0.15; IPL = 0.33 and CL = 0.17; IPL = 0.17, respectively). As expected, higher relative abundances of crenarchaeol increase discrepancies between the two RIs since crenarchaeol contributes four (cyclopentyl) rings to the RI with crenarchaeol. Adding crenarchaeol to the RI of MRCHSGNN093 increased CL RI from 2.71 to 3.06 and IPL from 3.09 to 3.73, while the RI of SMHNN007 increased

from 1.52 to 2.09 for CL and 1.25 to 1.88 for IPL. These results indicate that incorporating crenarchaeol into RI calculations must be thought through for springs with high concentrations of crenarchaeol.

Depending on the intended purpose of Ring Index, crenarchaeol may best be excluded from RI calculations. In marine sediments, RI can be used to determine whether the TEX_86_ temperature estimates are influenced by factors other than temperature, because Ring Index and TEX_86_ values have a global correlation when temperature is the controlling factor (Zhang et al., 2016). However, if variables other than temperature are more important for ring cyclization, then the relationship between Ring Index and TEX_86_ temperatures fails. Temperature is associated with crenarchaeol distributions, so including crenarchaeol in RI for paleothermometry may be reasonable even if crenarchaeol increases membrane permeability if it can provide a nuanced perspective into GDGT cyclization responses to temperature. If RI is being used to indicate cyclopentyl-forming enzyme activity, crenarchaeol may be included as a four-ringed member. However, if one is using RI as a stress response or indictor of decreased permeability, it may be best to exclude crenarchaeol from ring index, because this lipid prefers moderate temperatures, supporting a membrane-expanding function.
